# Romantic Relationship Quality and Eating Disorder Symptoms in Late Pregnancy: The Serial Mediating Role of Depression and Body Dissatisfaction

**DOI:** 10.3390/bs15101392

**Published:** 2025-10-14

**Authors:** Giulia Costanzo, Nadia Barberis, Eleonora Bevacqua, Maria Rita Infurna, Giorgio Falgares

**Affiliations:** 1 Department of Psychology, Educational Science, and Human Movement, University of Palermo, 90128 Palermo, Italy; giulia.costanzo01@unipa.it (G.C.); eleonora.bevacqua@unipa.it (E.B.); mariarita.infurna@unipa.it (M.R.I.); giorgio.falgares@unipa.it (G.F.); 2 Department of Health Sciences, University Magna Graecia of Catanzaro, 88100 Catanzaro, Italy

**Keywords:** romantic relationship quality, eating disorders, late pregnancy, third trimester, perinatal depressive symptoms, body dissatisfaction, maternal psychological well-being

## Abstract

Late pregnancy represents a critical period for the onset of eating disorder symptoms, particularly in the presence of psychological and relational vulnerabilities. Among these, the quality of the romantic relationship has received limited empirical attention, despite its potential role in shaping women’s psychological adjustment, influencing both mood and body image. The present study examined the association between romantic relationship quality and eating disorder symptoms during the third trimester of pregnancy, considering the mediating roles of depressive symptoms and body dissatisfaction. A sample of 231 Italian pregnant women (Mage = 32.3 years) completed four self-report measures: the Dyadic Adjustment Scale-7, the Edinburgh Postnatal Depression Scale, the Body Image in Pregnancy Scale, and the Eating Disorder Examination Questionnaire-Short. A serial mediation model was tested, including pre-pregnancy body mass index as a covariate. Results indicated that lower romantic relationship quality was associated with greater eating disorder symptoms through higher depressive symptoms and body dissatisfaction, which acted both independently and sequentially. These findings highlight the complex interplay between relational and psychological factors in the development of disordered eating during pregnancy, emphasizing the need for early screening and integrated interventions addressing both interpersonal and intrapersonal domains.

## 1. Introduction

Pregnancy constitutes a sensitive period characterized by profound physiological and psychosocial transformations that affect both expectant parents. In particular, during the third trimester (from 28 weeks of gestation to delivery), pregnant women and their partners are required to adjust to new responsibilities and demands, while beginning to negotiate childcare practices in preparation for childbirth (Baldoni et al., 2020). This developmental phase entails a significant reorganization within the couple, in which previous relational models and representations must be revised to accommodate the emerging family configuration (Zhi et al., 2025). Such reorganization may influence the quality of the romantic relationship, which, in turn, plays a pivotal role in shaping the emotional well-being of both partners (Yang et al., 2023).

Romantic relationship quality is a multidimensional construct that refers to individuals’ subjective evaluations of various aspects of couple functioning, including intimacy and cohesion, consensus, communication patterns, conflict resolution strategies, and overall relationship satisfaction (Roberson et al., 2018). Research has predominantly examined the impact of romantic relationship quality on pregnant women’s mental health, showing that high-quality relationships represent a crucial resource for promoting the expectant mother’s emotional well-being and for facilitating effective coping with pregnancy-related stressors (Antoniou et al., 2021; Daugherty et al., 2022). Conversely, low relationship quality has been associated with heightened psychological distress and maladaptive behaviors (Costa et al., 2020; Jonsdottir et al., 2017). These findings are theoretically grounded in recent developments within attachment theory. In adulthood, the romantic partner constitutes the primary attachment figure (Mikulincer & Shaver, 2007). Stressful circumstances—such as those emerging during pregnancy—tend to activate the attachment system, prompting the expectant mother to seek proximity and support from her partner as a means of alleviating anxiety and distress (Simpson & Rholes, 2017). However, when the romantic relationship is unsatisfactory, the partner may be perceived as unable to meet the pregnant woman’s attachment needs. Consequently, the attachment system remains chronically activated, emotional relief is not achieved, and psychological distress may ensue.

Importantly, romantic relationship quality should be understood within the broader context of gender dynamics. In heterosexual couples, relationship functioning is often shaped by prevailing gender norms and power imbalances, which can exert a significant influence on women’s psychological well-being (Habarth et al., 2019). In certain contexts, low relationship quality may not merely reflect limited intimacy or poor communication, but may instead conceal more severe relational dynamics, including physical or psychological violence (Shortt et al., 2010). Empirical evidence has underscored the high prevalence of coercion and abuse during pregnancy, as well as their detrimental effects on maternal mental health and overall quality of life (Howard et al., 2013). Although the present study did not directly assess these experiences, situating romantic relationship quality within this broader sociocultural framework is essential for a comprehensive understanding of the associations between relational variables and women’s emotional well-being.

Among studies examining the potential adverse effects of poor romantic relationship quality during pregnancy, several have explored its role in the emergence of eating disorder symptoms (EDS). Specifically, Baskin et al. (2020) found that lower relationship satisfaction was associated with higher levels of EDS during pregnancy, consistent with prior findings by Knoph Berg et al. (2011) and Gonçalves et al. (2015), which also identified links between poorer intimate relationship quality and dysfunctional eating behaviors before childbirth. As previously discussed, the romantic relationship serves as a primary attachment bond and a central mechanism for emotion regulation (Mikulincer & Shaver, 2007; Sousa-Gomes et al., 2022). When this relationship is unsatisfactory, its regulatory function may become impaired, leaving the pregnant woman more vulnerable to adopting alternative, maladaptive coping strategies to manage distress, including disordered eating behaviors (Haynos & Fruzzetti, 2011).

EDS encompass a broad spectrum of maladaptive cognitions and behaviors related to eating, weight, and body image—such as restrictive eating, binge-eating episodes, compensatory behaviors (e.g., self-induced vomiting, excessive exercise, fasting, or laxative use), and pervasive concerns with shape and weight (Bannatyne et al., 2018; Costanzo et al., 2025). During pregnancy, these symptoms have been associated with a range of adverse maternal and fetal outcomes, including an increased risk of gestational hypertension, miscarriage, preterm birth, low birth weight, and difficulties initiating breastfeeding (Sommerfeldt et al., 2022). Furthermore, research suggests that EDS may compromise the quality of maternal–fetal bonding and contribute to the development of perinatal affective disturbances, potentially hindering the transition to motherhood and disrupting early mother–infant interactions (Martini et al., 2022).

Building on this framework, a deeper understanding of the association between romantic relationship quality and EDS during pregnancy—and of the mechanisms underlying this relationship—appears crucial for preventing adverse pregnancy-related outcomes and informing interventions aimed at promoting maternal and fetal physical and psychological health.

Previous research has suggested that perinatal depressive symptoms may mediate the link between romantic relationship quality and EDS during pregnancy. Specifically, Baskin et al. (2020) found that poorer relationship quality was associated with greater disordered eating during mid-to-late pregnancy, and that this association was fully mediated by depressive symptoms. Poor romantic relationship quality is, indeed, a well-established psychosocial risk factor for depression, particularly during the perinatal period (Costa et al., 2020; Røsand et al., 2011; Yang et al., 2023). From an attachment-theoretical perspective, the romantic partner represents a primary source of emotional regulation in adulthood (Mikulincer & Shaver, 2007); when this regulatory function is compromised, vulnerability to depressive symptoms may increase. The transition to motherhood constitutes a critical period in which women particularly benefit from a caring, emotionally available, and responsive partner who can support them in coping with pregnancy-related challenges (Baskin et al., 2020). Conversely, poor relationship quality may heighten feelings of distress and loneliness, negatively affecting the expectant mother’s emotional well-being and increasing the likelihood of depressive symptoms (Yang et al., 2023). In turn, pregnant women may resort to dysfunctional emotion regulation strategies—such as disordered eating behaviors—to alleviate negative affect and mitigate depressive states (Costanzo et al., 2025). This notion is consistent with interpersonal theories of eating disorders, which propose that disordered eating behaviors may function as maladaptive coping strategies for managing negative emotions arising from relational difficulties and conflict (Ansell et al., 2012; Miniati et al., 2018).

Another mechanism potentially involved in the association between romantic relationship quality and antenatal EDS is body dissatisfaction. Pregnancy is a period marked by profound physical transformations, including weight gain, changes in body size and shape, and physical symptoms such as fatigue, back pain, swelling, and skin alterations (Linde et al., 2022). These changes become particularly pronounced during the third trimester, requiring women to reevaluate their body image and adjust to a new physical condition. However, a poor relationship with one’s partner may hinder this adjustment process and foster body dissatisfaction, defined as a subjective negative evaluation of one’s body or specific body parts (Chan et al., 2020). Although the association between romantic relationship quality and body dissatisfaction during pregnancy has not yet been directly examined, evidence from other populations (e.g., community samples and women recovering from breast cancer surgery) suggests that intimate relationship factors can substantially influence body image. Specifically, lower relationship quality has been associated with greater dissatisfaction with one’s physical appearance (Cairo Notari et al., 2017; Stiles et al., 2022). It is therefore plausible that a similar pattern could emerge during pregnancy, particularly in the later stages of gestation: a supportive and affectionate partner relationship may serve as a crucial resource that helps the pregnant woman adjust to bodily changes and feel secure in her evolving appearance, whereas a poor relationship may contribute to feelings of unattractiveness and diminished self-worth, making body acceptance more difficult. Heightened body dissatisfaction may, in turn, increase vulnerability to maladaptive compensatory behaviors—such as restrictive dieting or fasting—which can represent early indicators of prenatal disordered eating (Fuller-Tyszkiewicz et al., 2020; Vanderkruik et al., 2022). Individual factors may also shape how women experience bodily changes during pregnancy. In particular, previous research has consistently shown that a higher pre-pregnancy body mass index (BMI) is associated with greater body dissatisfaction and increased vulnerability to EDS during gestation (Baskin et al., 2021; Chan et al., 2020). Pre-pregnancy BMI may therefore represent a critical variable to consider when investigating pregnant women’s adjustment to body changes and their risk for disordered eating behaviors.

Based on the above, depressive symptoms and body dissatisfaction may constitute two independent psychological mechanisms leading to prenatal EDS, starting from a condition of relational suffering. However, these two mechanisms may also be interconnected, operating in a sequential manner. Specifically, depressive states arising from a poor relationship with one’s partner may hinder the woman’s ability to adapt to pregnancy-related bodily changes, thereby fostering dissatisfaction with her physical appearance. Previous studies have examined the hypothesis of a direct association between depressive symptoms and prenatal body dissatisfaction (Silveira et al., 2015). For instance, Clark et al. (2009) found that depressive symptoms during late pregnancy significantly predicted several dimensions of body dissatisfaction, including perceptions of feeling fatter, less attractive, and less strong or fit. Depressive states may indeed heighten the tendency to evaluate oneself negatively across multiple domains, including body image, and to selectively focus attention on the most disliked body areas, thereby amplifying body dissatisfaction (Chan et al., 2020). As discussed above, this heightened dissatisfaction may, in turn, promote the adoption of maladaptive eating behaviors and increase vulnerability to EDS, as supported by previous research highlighting the role of body image disturbances in the development of perinatal disordered eating (Vanderkruik et al., 2022).

Nevertheless, further research is needed to clarify the relationship between depressive symptoms and body dissatisfaction during pregnancy, as well as their potential role as serial mediators in the association between romantic relationship quality and antenatal EDS.

### The Present Study

Building on the aforementioned theoretical and empirical considerations, the present study aimed to examine the association between romantic relationship quality and EDS in a sample of women in the third trimester of pregnancy. Specifically, we investigated the potential mediating roles of prenatal depressive symptoms and body dissatisfaction in this relationship. Grounded in attachment theory and prior evidence linking poor relationship quality to both affective distress and body image disturbances during pregnancy, we formulated the following hypotheses:

**Hypothesis** **1.** *Depressive symptoms would mediate the association between romantic relationship quality and EDS.*

**Hypothesis** **2.** *Body dissatisfaction would mediate the association between romantic relationship quality and EDS.*

**Hypothesis** **3.** *Depressive symptoms and body dissatisfaction would operate as serial mediators in the association between romantic relationship quality and EDS. Specifically, lower relationship quality would be associated with greater depressive symptoms, which in turn would predict increased body dissatisfaction, ultimately leading to higher levels of EDS.*

## 2. Method

### 2.1. Participants and Procedure

The Bioethics Committee of the University of Palermo (protocol number: 167/2023; ethics approval date: 11 February 2023) approved this study. All procedures were conducted in accordance with the ethical principles for psychological research, following the Declaration of Helsinki and its revisions (World Medical Association, 2001), as well as the ethics guidelines of the American Psychological Association (2010).

A total of 231 Italian heterosexual pregnant women (aged 20–45, Mage = 32.3, *SD* = 4.7) participated in the study. Participants were recruited from obstetrics and gynecology units of hospitals and birth centers in Southern Italy. This was a cross-sectional, multicenter, and descriptive study. Data collection was conducted between March and June 2024.

Inclusion criteria were (a) being in the third trimester of pregnancy, (b) being at least 18 years old, (c) currently being involved in a romantic relationship, and (d) being able to read, understand, and speak Italian. Exclusion criteria were (a) having a current or past clinical diagnosis of an eating disorder, (b) having a medical condition or pregnancy complication that could significantly affect psychological functioning (e.g., high-risk pregnancy, severe preeclampsia, hospitalization), and (c) inability or unwillingness to provide informed consent.

All participants received detailed information about the aims of the study, the voluntary nature of their participation, and the anonymity of their responses. Written informed consent was obtained prior to data collection.

Detailed sociodemographic characteristics and pregnancy-related information are reported in Table 1.

### 2.2. Measures

#### 2.2.1. Dyadic Adjustment Scale 7

The Dyadic Adjustment Scale–Short Form (DAS-7; Hunsley et al., 2001) was used to assess participants’ romantic relationship quality. Together with its extended version, the 32-item Dyadic Adjustment Scale (Gentili et al., 2002; Spanier, 1976), the DAS-7 is widely employed in both research and clinical settings due to its ability to capture the core dimensions of intimate relationship functioning (e.g., agreement, cohesion, satisfaction) and their effects on psychological well-being (Başgöl et al., 2024). The scale consists of 7 items (e.g., *“How often would you say you and your partner calmly discuss something together?”*; *“Please indicate the approximate extent of agreement or disagreement between you and your partner for each area: philosophy of life, aims and goals, amount of time spent together”*). The first six items are rated on a 6-point Likert scale ranging from 0 (*always disagree* or *never*) to 5 (*always agree* or *all the time*), whereas the last item—assessing overall happiness with the romantic relationship—is rated on a 7-point Likert scale from 0 (*extremely unhappy*) to 6 (*perfectly happy*). A total DAS-7 score was obtained by calculating the mean of all items, with higher scores indicating greater relationship quality. In the present study, internal consistency was acceptable (Cronbach’s α = 0.74).

#### 2.2.2. Edinburgh Postnatal Depression Scale

The Edinburgh Postnatal Depression Scale (EPDS; Benvenuti et al., 1999; Cox et al., 1987) was administered to assess depressive symptoms among pregnant women. Although originally developed to evaluate postnatal depression, the EPDS has also been validated for use during pregnancy (Kozinszky & Dudas, 2015). The EPDS includes 10 self-report items rated on a 4-point Likert scale (0–3). The total score was computed by averaging all items, with higher scores reflecting more severe depressive symptoms. In the present study, the EPDS showed good internal consistency (Cronbach’s α = 0.84).

#### 2.2.3. Body Image in Pregnancy Scale

The Body Image in Pregnancy Scale (BIPS; Watson et al., 2017) was used to assess body image during pregnancy. This 36-item self-report questionnaire measures seven dimensions: (1) preoccupation with physical appearance, (2) dissatisfaction with strength-related aspects of one’s body, (3) dissatisfaction with complexion, (4) sexual attractiveness, (5) prioritization of appearance over function, (6) appearance-related behavioral avoidance, and (7) dissatisfaction with specific body parts. Items are rated on a 5-point Likert scale ranging from 1 to 5. The total BIPS score was computed by averaging all items, with higher scores indicating greater body image disturbance. In this study, the BIPS demonstrated excellent internal consistency (Cronbach’s α = 0.90).

#### 2.2.4. Eating Disorder Examination–Questionnaire Short

The Eating Disorder Examination–Questionnaire Short (EDE-QS; Gideon et al., 2016) was used to evaluate eating disorder symptoms (EDS) over the past seven days. The EDE-QS is a 12-item self-report measure derived from the 28-item Eating Disorder Examination–Questionnaire (Calugi et al., 2017; Fairburn & Beglin, 1994). Each item is rated on a 4-point Likert scale (0–3). A total score was calculated by averaging all items, with higher scores indicating greater severity of EDS. The EDE-QS demonstrated good internal consistency in the present study (Cronbach’s α = 0.85).

### 2.3. Data Analysis

Statistical analyses were conducted using IBM SPSS Statistics version 20.0. Firstly, the Variance Inflation Factor (VIF) was used to assess multicollinearity. Descriptive statistics, including means (M), standard deviations (SD), and range, were then computed for all key study variables. Pearson correlation analysis was used to explore bivariate associations among romantic relationship quality (DAS-7), depressive symptoms (EPDS), body dissatisfaction (BIPS), and EDS (EDE-QS).

**Figure 1:**
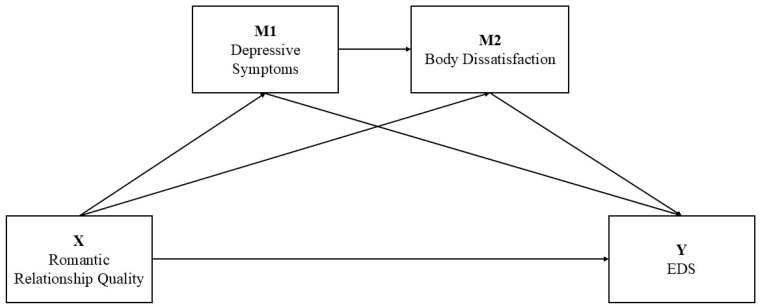
Proposed Mediation Model.

We employed PROCESS macro-Model 6 (Hayes, 2013) to test the hypothesized mediation model. Specifically, we examined the indirect pathways from romantic relationship quality to EDS through the mediation of antenatal depressive symptoms and body dissatisfaction (Figure 1). The significance of indirect effects was assessed using a bias-corrected bootstrap method with 5000 resamples. If the 95% confidence interval (CI) for the bootstrapped estimate did not include zero, then the indirect effect was considered significant at *p* < 0.05. Pre-pregnancy BMI was included in the model as a covariate to account for potential confounding effects.

## 3. Results

### 3.1. Testing for Multicollinearity

Collinearity diagnostics showed that the VIF for romantic relationship quality, depressive symptoms, body dissatisfaction, and pre-pregnancy BMI were 1.23, 1.36, 1.37, and 1.07, respectively. These values are well below the commonly accepted threshold of 5 (Hair et al., 2017), indicating no evidence of multicollinearity among the study variables.

### 3.2. Preliminary Analysis

Means (M), standard deviations (SD), range, skewness, and kurtosis of the study variables are presented in Table 2. We adopted Kline’s (2011) criteria of skewness < ±3 and kurtosis < ±10 to identify deviations from normality. No severe violation of univariate normality was detected, with the skewness values ranging from −1.10 to 1.41 and the kurtosis values ranging from 0.06 to 2.66. Pearson correlations among the study variables are also reported in Table 2. EDS were significantly and positively related to depressive symptoms (*r* = 0.36, *p* < 0.01) and to body dissatisfaction (*r* = 0.57, *p* < 0.01), whereas significant and negative associations were found with romantic relationship quality (*r* = −0.18, *p* < 0.01). Romantic relationship quality was significantly and negatively related also to depressive symptoms (*r* = −0.39, *p* < 0.01) and to body dissatisfaction (*r* = −0.33, *p* < 0.01). Depressive symptoms and body dissatisfaction were significantly and positively associated (*r* = 0.44, *p* < 0.01).

### 3.3. Mediation Analysis

A mediation model was tested to explore whether prenatal depressive symptoms (M1) and body dissatisfaction (M2) mediated the association between romantic relationship quality (X) and EDS (Y) during pregnancy, with pre-pregnancy BMI included as a covariate.

As shown in Table 3, results revealed a significant negative effect of romantic relationship quality on prenatal depressive symptoms, so that higher depressive symptoms were predicted by lower relationship quality (β = −0.39, *SE* = 0.05, *p* < 0.001). Depressive symptoms, in turn, had a significant positive effect on body dissatisfaction (β = 0.36, *SE* = 0.07, *p* < 0.001). Results also showed a significant negative effect of romantic relationship quality on body dissatisfaction, so that higher dissatisfaction was predicted by lower relationship quality (β = −0.20, *SE* = 0.05, *p* < 0.001). Finally, both prenatal depressive symptoms (β = 0.16, *SE* = 0.06, *p* < 0.01) and body dissatisfaction (β = 0.48, *SE* = 0.05, *p* < 0.001) emerged as significant predictors of EDS. Conversely, the direct effect of romantic relationship quality on EDS was found non-significant in the presence of the mediators (β = 0.04, *SE* = 0.04, *p* = 0.515), indicating a full mediation of depressive symptoms and body dissatisfaction in the association between romantic relationship quality and EDS during pregnancy. The final model was significant with *R*^2^ = 0.357, *F* (4, 226) = 31.423, *p* < 0.001.

**Figure 2:**
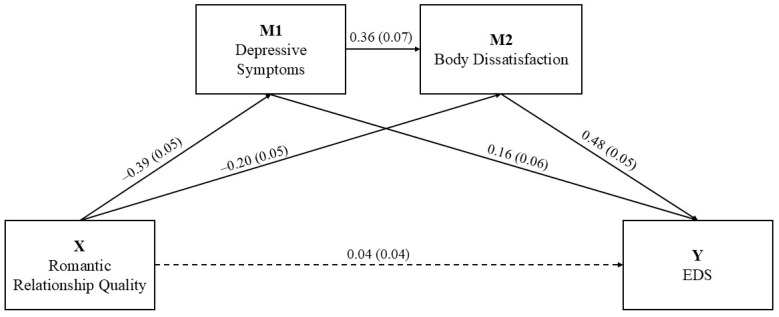
The serial mediation model of depressive symptoms and body dissatisfaction in the relationship between romantic relationship quality and ED symptoms. Note: The values represent the standardized coefficients with standard error in parentheses. The solid lines represent the significant paths for *p* < 0.05. The dashed lines represent the non-significant paths.

Bootstrapping analyses with 5000 resamples confirmed the significance of all indirect effects (Table 4). Specifically, the indirect effect of romantic relationship quality on EDS via depressive symptoms alone was significant (β = −0.06, BootSE = 0.03), as well as the indirect path via body dissatisfaction alone (β = −0.10, BootSE = 0.03). The sequential pathway through both depressive symptoms and body dissatisfaction was also significant (β = −0.07, BootSE = 0.02), supporting a serial mediation model in which lower romantic relationship quality predicts higher EDS during pregnancy through higher depressive symptoms and increased body dissatisfaction. The detailed pathway model is shown in Figure 2.

Pre-pregnancy BMI was included in the model as a covariate to control its potential influence on the study’s variables. While it did not show a significant association with depressive symptoms (β = 0.01, *p* = 0.842), it emerged as a significant predictor of both body dissatisfaction (β = 0.22, *p* < 0.001) and EDS (β = 0.13, *p* < 0.05), so that higher BMI was associated with greater body dissatisfaction and increased EDS. However, the significance of the indirect effects remained unchanged when controlling for BMI, indicating that the mediation pathways from romantic relationship quality to EDS through depressive symptoms and body dissatisfaction were maintained besides the potential effects of pre-pregnancy BMI.

## 4. Discussion

This study aimed to examine the association between romantic relationship quality and EDS during the third trimester of pregnancy, considering the potential mediating roles of prenatal depressive symptoms and body dissatisfaction.

Consistent with our first hypothesis, results showed that prenatal depressive symptoms mediated the association between romantic relationship quality and EDS. Specifically, a poorer relationship with one’s partner was associated with higher depressive symptoms, which in turn were linked to greater EDS. This finding aligns with attachment theory (Mikulincer & Shaver, 2007) and interpersonal models of psychopathology (Ansell et al., 2012; Miniati et al., 2018), as well as with previous empirical evidence (Baskin et al., 2020), suggesting that a lack of a satisfying intimate relationship may have detrimental consequences on maternal mental health, particularly in late pregnancy. The third trimester represents a critical developmental phase characterized by heightened physical, hormonal, and psychosocial demands, in which women are required to renegotiate their identity and prepare for motherhood. Within this vulnerable context, the absence of a supportive romantic bond may diminish emotional resources, increase perceived stress, and intensify feelings of isolation and inadequacy (Costa et al., 2020; Yang et al., 2023). Consequently, depressive symptoms may emerge and increase the likelihood of engaging in maladaptive eating behaviors as an attempt to regulate or suppress negative affect and obtain temporary relief (Costanzo et al., 2025). From this perspective, EDS during pregnancy may serve an emotion-regulation function in response to relational and psychological distress.

Our second hypothesis was also supported: body dissatisfaction mediated the relationship between romantic relationship quality and EDS. Specifically, lower romantic relationship quality was associated with higher body dissatisfaction, which in turn predicted greater EDS. This finding extends prior research linking romantic relationship quality to body image (Cairo Notari et al., 2017; Stiles et al., 2022), demonstrating that this association is also relevant in the late stages of pregnancy. Romantic relationships may play a crucial role in shaping women’s perceptions of their changing bodies during gestation (Chan et al., 2020). A positive, supportive relationship can facilitate the acceptance of pregnancy-related bodily changes, serving as a protective factor against body image concerns. Conversely, relational dissatisfaction may heighten sensitivity to perceived flaws and foster self-critical attitudes toward one’s appearance. Within this context, EDS may function as maladaptive strategies aimed at alleviating body-related distress, regaining control, or conforming to perceived standards of attractiveness. This interpretation is consistent with previous studies identifying body dissatisfaction as one of the most robust and proximal risk factors for disordered eating (Fuller-Tyszkiewicz et al., 2020; Vanderkruik et al., 2022).

Third, we hypothesized that depressive symptoms and body dissatisfaction would operate sequentially as mediators in the link between romantic relationship quality and EDS. Findings supported this serial mediation model: poor romantic relationship quality was associated with higher depressive symptoms, which predicted greater body dissatisfaction, ultimately leading to more severe EDS. In line with prior research (Clark et al., 2009), these results suggest that psychological distress—in the form of depressive symptoms—may act as a key antecedent of body image disturbances by reinforcing attentional biases toward disliked body parts and negative self-evaluations (Chan et al., 2020). As pregnancy progresses, these distortions may intensify, particularly in the presence of relational suffering, resulting in elevated body dissatisfaction, a well-established proximal risk factor for EDS (Fuller-Tyszkiewicz et al., 2020).

This serial mediation pathway underscores the complex interplay between relational, emotional, and body-related factors in understanding EDS during pregnancy. Romantic relationship difficulties may operate as distal interpersonal stressors, eliciting depressive symptoms that erode body image, thereby increasing vulnerability to disordered eating. The absence of a significant direct effect of romantic relationship quality on EDS, once the mediators were included, indicates a full mediation pattern. Although the cross-sectional nature of the data precludes causal inference, this finding suggests that relational suffering may not directly influence disordered eating; rather, it promotes an affective and cognitive vulnerability—marked by depression and body dissatisfaction—that predisposes women to EDS. This perspective reinforces the conceptualization of EDS during pregnancy as a multifactorial condition shaped by interrelated interpersonal and psychological processes. Importantly, these processes are embedded in a broader sociocultural context, which shapes the way intimate relationships, emotions, and body image are experienced. Specifically, Italy is a collectivistic country, with high familism values and elevated interdependence among family members (Rodríguez-González et al., 2020). In such context, family bonds—including the one with the romantic partner—are fundamental for the definition of one’s personal identity and for fostering (or hindering) emotional well-being during key transitional periods such as pregnancy (Donati, 2003). At the same time, Italian and Western cultural ideas place a significant emphasis on beauty, thinness, and physical appearance, which may predispose women to negative feelings toward perinatal body transformations (Grano et al., 2025). Cultural norms may therefore amplify the relevance of romantic relationship quality in influencing mood and body image during pregnancy, as well as the salience of body concerns in predicting patterns of disordered eating.

Finally, pre-pregnancy BMI was included as a covariate due to its established association with both body dissatisfaction and EDS (Baskin et al., 2021; Chan et al., 2020). Consistent with expectations, BMI correlated significantly with both constructs. However, its inclusion did not attenuate the significance or magnitude of the hypothesized mediation pathways, suggesting that the observed associations are not merely a reflection of pre-existing weight status but represent psychologically meaningful processes specific to the perinatal period. Thus, while BMI remains a relevant background characteristic, it does not account for the complex interplay among relational functioning, emotional suffering, body image concerns, and eating behaviors observed during pregnancy.

### 4.1. Limitations

Our findings should be considered within the context of some limitations. First, the cross-sectional nature of this research does not allow for the examination of causal relationships among the study variables. Future research would benefit from adopting longitudinal designs to assess whether changes in EDS during pregnancy are influenced by variations in relational satisfaction, mood, and body image across time. Second, all constructs were assessed using self-report measures, which may be susceptible to social desirability biases and could have inflated some of the observed associations. Future research should adopt multi-method approaches, integrating self-report instruments with qualitative interviews or clinician-administered assessments, to provide a more comprehensive understanding of the studied phenomena. Third, the EDE-QS was not specifically designed to evaluate EDS in pregnant populations. Nevertheless, it has been previously employed in Italian pregnant samples (Costanzo et al., 2025), and its extended version, the EDE-Q, has demonstrated solid psychometric properties in this research field (Stephens et al., 2024). Future investigations could benefit from the development or validation of pregnancy-specific measures of disordered eating that better capture gestational changes in eating attitudes and behaviors. Fourth, this study did not account for past depression or experiences of gender-based violence, particularly psychological coercion or abuse. This represents a key limitation, as exposure to severe relational harm could partially explain some of the observed associations between romantic relationship quality and the psychological variables under investigation. Future studies should therefore include measures assessing both lifetime depressive episodes and intimate partner violence to clarify their role in shaping perinatal mental health outcomes. Fifth, the sample consisted exclusively of heterosexual pregnant women, which may limit the generalizability of the findings to other populations (e.g., sexual minority women or gender-diverse individuals). Future research should address this issue by recruiting larger and more diverse samples to capture the heterogeneity of experiences across different gender identities and sexual orientations. Sixth, as the sample was composed entirely of Italian women, cultural specificities may have influenced the interplay among interpersonal and intrapersonal variables. Consequently, the generalizability beyond the Italian context is limited. Future studies should replicate and extend this model across diverse sociocultural backgrounds to determine whether the observed pathways are culturally specific or reflect more universal processes. Another limitation concerns the use of BMI as a covariate. Although BMI is a widely used indicator of body fat in adults, it has well-documented limitations, such as its inability to distinguish between fat and muscle mass (Bray, 2023) and its poor sensitivity to variations in body shape and fat distribution across genders and ethnic groups, which may introduce gendered and racial biases (Sweatt et al., 2024). Finally, this study did not include sociocultural variables (e.g., internalization of appearance ideals, perceived social pressures regarding body shape) that are known to affect body image and EDS in both general and perinatal populations (da Silva et al., 2020; Pedersen et al., 2018). Future research should incorporate validated measures of these constructs for a deeper understanding of the sociocultural pathways contributing to disordered eating during pregnancy.

### 4.2. Strengths and Practical Implications

Notwithstanding these limitations, this is the first study to explore the serial mediation of depressive symptoms and body dissatisfaction in the association between romantic relationship quality and EDS during late pregnancy. A key strength of this research lies in its contribution to a more nuanced understanding of perinatal disordered eating as a multifaceted phenomenon shaped by the interaction between relational and psychological dimensions. Another strength is the specific focus on the third trimester of pregnancy, a period of heightened vulnerability in the perinatal process. As childbirth approaches, profound couple and family reorganizations are required (Zhi et al., 2025), potentially affecting relationship quality and women’s emotional well-being. At the same time, physical transformations become more pronounced during this stage (Linde et al., 2022), which may render the acceptance of bodily changes increasingly challenging. Late pregnancy therefore represents a critical window for the identification of risk factors associated with the emergence or exacerbation of EDS.

Findings from this study could also have important practical—preventive and clinical—implications. From a preventive standpoint, perinatal healthcare providers (e.g., gynecologists, obstetricians, midwives, general practitioners) could benefit from incorporating comprehensive screening protocols for relational stressors, depressive symptoms, and body image concerns into routine prenatal visits. Such practices would facilitate early detection of psychosocial vulnerability for EDS, which is essential when addressing maternal mental health. Indeed, early identification of at-risk women would allow to promote timely, targeted interventions aimed at preventing the onset or exacerbation of maladaptive eating behaviors, which may lead to serious complications for both the mother and the fetus (Sommerfeldt et al., 2022). In this regard, interdisciplinary collaboration between healthcare providers and mental health specialists may be essential to design integrated care programs addressing both medical and psychosocial aspects of maternal health.

From a clinical perspective, results from this study suggest that psychological interventions should aim to support the couple’s accommodation to pregnancy-related demands by promoting more adaptive relational dynamics, including greater intimacy, emotional cohesion, and more constructive communication. These aspects are especially relevant during the third trimester, when the imminent transition to parenthood requires a profound reorganization of roles, expectations, and family functioning (Zhi et al., 2025). Moreover, partner-inclusive programs or couple-based interventions could be particularly beneficial, as they have the potential to foster relational stability and emotional security, which could exert positive effects on the woman’s psychological well-being and on her acceptance of body changes (Stapleton et al., 2012).

Finally, findings could have important implications at a policy level, supporting the integration of psychosocial assessment into perinatal care guidelines to ensure a more holistic approach to maternal and fetal health.

## 5. Conclusions

This study contributes to a better understanding of the association between romantic relationship quality and EDS during the late stages of pregnancy. Results suggest that this association is mediated by depressive symptoms and body dissatisfaction, which may operate both independently and in a sequential manner.

Future longitudinal studies are warranted to confirm the directionality of these associations and to evaluate whether interventions targeting both interpersonal and intrapersonal mechanisms can mitigate the risk of EDS across the perinatal period, ultimately improving both maternal and infant outcomes. Moreover, dyadic designs including both partners may yield valuable insights into how relationship processes influence maternal adjustment and vulnerability to disordered eating. Finally, future research should also consider cross-cultural replications and the inclusion of additional psychosocial risk factors, such as gender-based violence and sociocultural variables, to ensure a more comprehensive understanding of the determinants of disordered eating during pregnancy.

## Figures and Tables

**Table 1:** Sociodemographic and Pregnancy-Related Information.

Variable	n	%
Educational Level		
Middle school diploma	42	18.1
High school diploma	72	31.2
Bachelor’s/Master’s degree	69	29.9
Post-Lauream degree	48	20.8
Work Status		
Housewife/Unemployed	92	39.8
Occasional worker	27	11.7
Employed	112	48.5
Income		
Low	21	9.1
Medium	144	62.3
High	66	28.6
Planned Pregnancy		
Yes	156	67.5
No	75	32.5
Primiparous		
Yes	139	60.2
No	92	39.8
Medically Assisted Procreation		
Yes	11	4.8
No	220	95.2
Pre-Pregnancy Body Mass Index		
Underweight (<18.5)	23	10.0
Normal weight (18.5–24.9)	131	56.7
Overweight (25.0–29.9)	52	22.5
Obesity (>30.0)	25	10.8

**Table 2:** Correlation Matrix, Means, Standard Deviations, Range, Skewness, and Kurtosis of the Study Variables.

	1	2	3	4	5
1. Romantic Relationship Quality	--				
2. Depressive Symptoms	−0.39 **	--			
3. Body Dissatisfaction	−0.33 **	0.44 **	--		
4. EDS	−0.18 **	0.36 **	0.57 **	--	
5. Pre-Pregnancy BMI	0.04	−0.01	0.21 **	0.23 **	--
*M*	4.23	0.55	2.29	0.52	24.13
*SD*	0.69	0.51	0.57	0.48	5.47
Range	1.54–5.14	0–2.50	1.06–4.00	0–2.67	14.7–45.5
*Skewness*	−1.10	1.14	0.42	1.14	1.41
*Kurtosis*	1.71	1.13	0.06	1.22	2.66

Note: *N* = 231. ** *p* < 0.01.

**Table 3:** The serial mediation analyses.

Outcome	Predictors	*R* ^2^	*F*	β	*t*	*p*	95% CI
Depressive Symptoms	Romantic Relationship Quality	0.153	20.526	−0.39	−6.41	<0.001	−0.38, −0.20
	Pre-Pregnancy BMI			0.01	0.20	0.842	−0.01, 0.01
Body Dissatisfaction	Romantic Relationship Quality	0.271	28.083	−0.20	−3.19	<0.001	−0–26, −0.06
	Depressive Symptoms			0.36	5.88	<0.001	0.27, 0.54
	Pre-Pregnancy BMI			0.22	3.91	<0.001	0.01, 0.04
EDS	Romantic Relationship Quality	0.357	31.423	0.04	0.65	0.515	−0.05, 0.11
	Depressive Symptoms			0.16	2.61	<0.01	0.04, 0.27
	Body Dissatisfaction			0.48	7.75	<0.001	0.31, 0.52
	Pre-Pregnancy BMI			0.13	2.34	<0.05	0.01, 0.02

Note: Pre-Pregnancy BMI was included in the model as a covariate. Standardized regression coefficients (β) are reported. 95% CI = 95% confidence interval.

**Table 4:** Total, direct, and indirect effects of romantic relationship quality (X) on EDS (Y) through depressive symptoms (M1) and body dissatisfaction (M2).

	Effect	BootSE	Bootstrapping 95% CI
Total effect	−0.13	0.04	−0.22, −0.05
Direct effect	0.03	0.05	−0.07, 0.11
Indirect effect (X→M1→Y)	−0.06	0.03	−0.12, −0.01
Indirect effect (X→M2→Y)	−0.10	0.03	−0.16, −0.03
Indirect effect (X→M1→M2→Y)	−0.07	0.02	−0.11, −0.03

Note: All effects are adjusted for Pre-Pregnancy BMI, included as a covariate in the model. Effect = standardized regression coefficient. BootSE = bootstrap standard error. Bootstrapping 95% CI = 95% bias-corrected bootstrap confidence interval, based on 5000 resamples.

## Data Availability

The data presented in this study are available upon reasonable request from the corresponding author.
